# Contribution to the "Atlas of the Russian Flora": Twelve local floras of European Russia

**DOI:** 10.3897/BDJ.9.e73013

**Published:** 2021-09-15

**Authors:** Alexey P. Seregin

**Affiliations:** 1 M.V. Lomonosov Moscow State University, Moscow, Russia M.V. Lomonosov Moscow State University Moscow Russia

**Keywords:** flora, Russia, tracheophytes, dataset, data paper, observations

## Abstract

**Background:**

The purpose of this dataset is to deliver to a wider audience in the form of GBIF-mediated data vast floristic materials collected by the author across various localities of European Russia from 2001-2019 (Arkhangelsk, Tver, Vladimir, Tula, Lipetsk, Voronezh Oblasts, Krasnodar Krai, City of Moscow and Komi Republic). Taxonomic data on vascular plants for ten locations were mobilised from the papers and technical reports published in Russian and standardised. Floristic treatments for two locations (Yasnaya Polyana and Tsaritsyno) have never been published before.

**New information:**

The newly-prepared dataset includes 5,309 species records, i.e. one species record per each local flora. These are either native or alien (fully naturalised and casual) species. All records within one local flora have the same centroid coordinates and coordinate uncertainty in metres. Floristic inventories from the following locations were mobilised: 01. Ustya, Arkhangelsk Oblast (543 species, 1,500 km^2^); 02. Zaseki, Tula Oblast (593 species, 60 km^2^); 03. Polibino, Lipetsk Oblast (553 species, 70 km^2^); 04. Khrenovoye, Voronezh Oblast (665 species, 200 km^2^); 05. Troyeruchitsa, Tver Oblast (501 species, 10 km^2^); 06. Man-Pupu-Ner, Komi Republic (182 species, ca. 300 km^2^); 07. Middle Lyaga, Komi Republic (143 species, ca. 300 km^2^); 08. Utrish, Krasnodar Krai (933 species, 195 km^2^); 09. Yasnaya Polyana, Tula Oblast (236 species, 2.2 km^2^); 10. Bogolyubovsky Lug, Vladimir Oblast (289 species, 1.7 km^2^); 11. Tsaritsyno, City of Moscow (359 species, 5.3 km^2^); 12. Patakino, Vladimir Oblast (312 species, 1.1 km^2^). According to the GBIF taxonomic backbone, the dataset covers 1,806 species, 669 genera and 127 families of tracheophytes.

## Introduction

For the last 20 years, the author thoroughly studied under various circumstances vascular plants in several locations across European Russia. These contributions include twelve local floras — five places of summer field trips of Lomonosov Moscow State University students (Ustya, Zaseki, Polibino, Khrenovoye and Utrish) and seven locations in legally-protected areas (Man-Pupu-Ner and Middle Lyaga in Pechoro-Ilych Reserve, Troyeruchitsa, Yasnaya Polyana, Bogolyubovsky Lug, Tsarirsyno and Patakino). This is a step towards the "Atlas of the Russian Flora" currently prepared under my supervision.

### 01. Ustya

This local flora covers the largest area ca. 1,500 km^2^. It is situated in the taiga zone (boreal evergreen conifer forests) along the southern border of Arkhangelsk Oblast. The primary mobilised source (Seregin and Goryainova 2003) included 544 numbered species. Five species known only from Chadroma, a distant locality on the NW corner of the studied area, were excluded to reduce coordinate uncertainty from 38,000 m to a reasonable 21,000 m. Four unnumbered *Pilosella* hybrids were included into the dataset, which finally lists 543 entries.

The Ustya flora is well documented by vouchers deposited at MWG Herbarium (Herbarium of Biogeography Department, Faculty of Geography, Lomonosov Moscow State University), including 232 specimens collected by the author in 2001 and 149 specimens in 2002 (collector's numbers from N-1 to N-380), as well as earlier collections by the department's staff. Unfortunately, there are no digitisation activities in this Herbarium at the moment.

### 02. Zaseki

This local flora (60 km^2^) was studied around the permanent camping site of the summer field practice of Faculty of Soil Science, Lomonosov Moscow State University. It is situated in the broadleaved forest zone (temperate deciduous hardwood forests) in Shchekino District, Tula Oblast. Two published sources were mobilised (Seregin 2011, Seregin 2015) which include 520 species discovered from 2005-2010 and an additional 73 species from 2011-2013. The centroid of the area is located in the camping site with proposed coordinate uncertainty 8,000 m.

The Zaseki flora is poorly documented by vouchers with the focus on common plants intensively collected by the students. The main herbarium set by the author is deposited at MHA Herbarium (Skvortsov Herbarium of Tsitsin Main Botanical Garden, Russian Academy of Sciences, Moscow). Only 15% of Eastern Europe holdings of this herbarium have been digitised so far (Seregin and Stepanova 2020); therefore, related occurrences from the MHA GBIF-mediated dataset (Seregin and Stepanova 2021) include only 85 specimens of 76 species collected from 2005-2013.

### 03. Polibino

This local flora (70 km^2^) was also studied around the permanent camping site of the summer field practice of Faculty of Soil Science, Lomonosov Moscow State University. It is situated in the forest-and-steppe zone (an ecotone between temperate deciduous hardwood forests and dry temperate grasslands) in Dankov District, Lipetsk Oblast along the Don River. Two published sources were mobilised (Seregin 2011, Seregin 2015) which include 506 species discovered from 2005-2010 supplemented by 50 species found from 2011-2013. The centroid of the area is located in the camping site with proposed coordinate uncertainty 7,000 m. Three misidentified species from the first source (Seregin 2011) were excluded from the dataset, which finally lists 553 entries.

The Polibino flora is unevenly documented by vouchers with focus on common plants and species of grasslands intensively collected by the students. The main herbarium set by the author is deposited at MW Herbarium (Herbarium of Lomonosov Moscow State University). This Herbarium is completely digitised at the moment and fully available online (Seregin 2018), but related occurrences from the MW GBIF-mediated dataset (Seregin 2021b) include only 331 specimens of 256 species collected in this area from 2005-2013.

### 04. Khrenovoye

This local flora (200 km^2^) was studied within the borders of the northern part of Khrenovskoy Bor (Khrenovoye Pine Forest) near the permanent camping site of the summer field practice of Faculty of Soil Science, Lomonosov Moscow State University. It is situated on southern edge of the forest-and-steppe zone (an ecotone between temperate deciduous hardwood forests and dry temperate grasslands) in Bobrov District, Voronezh Oblast along the Bityug River. The mobilised primary source (Seregin 2015) includes 665 species discovered from 2005-2013. The centroid of the area is located in the middle of the pine forest with proposed coordinate uncertainty 16,000 m.

The Khrenovoye flora is poorly documented by vouchers, because the student excursions are not allowed to enter the pine forest in a fire-risk summer period. The main herbarium set by the author is deposited in MW Herbarium (Herbarium of Lomonosov Moscow State University). The related occurrences from the MW GBIF-mediated dataset (Seregin 2021b) include 263 specimens of 228 species collected here from 2005-2013.

The flora of isolated pine forests of Voronezh Oblast is extraordinarily rich. For instance, Starodubtseva (1999) encounted 1,032 species for Usmansky Pine Forest near Voronezh (ca. 700 km^2^). Tzvelev (1988) listed ca. 1,200 species for Khopersky Nature Reserve (162 km^2^) located in the Khopersky Pine Forest. Kin and Starodubtseva (2012) included 802 species into the checklist of Khrenovoye Pine Forest. This dataset documents the flora of the northern part (roughly a half) of Khrenovoye Pine Forest.

### 05. Troyeruchitsa

This local flora (10 km^2^) was studied within the borders of the Troyeruchitsa State Nature Reserve and three adjacent settlements. It is situated in the taiga zone (boreal evergreen conifer forests) in Ostashkov District, Tver Oblast along the eastern coast of Lake Seliger. The mobilised primary source (Seregin 2020) includes 501 species recorded from 2016-2019, i.e. 456 species records from the Reserve and 45 records along its borders. The centroid of the area is located on the southern coast of Lake Beloye with proposed coordinate uncertainty 3,000 m.

The Troyeruchitsa flora was not documented properly by herbarium vouchers. The related occurrences from the MW GBIF-mediated dataset (Seregin 2021b) include only 63 specimens of 51 species of special interest collected by the author from 2016-2019. Additionally, a dataset of georeferenced photos made in 2019 as part of the Troyeruchitsa Local Flora project on iNaturalist accounts for 434 observations of 358 species. These photo vouchers are available on GBIF (Ueda 2021) in line with the "Flora of Russia" initiative (Seregin et al. 2020).

### 06. Man-Pupu-Ner & 07. Middle Lyaga

These two local floras (ca. 300 km^2^) were studied within the borders of Man-Pupu-Ner and Middle Lyaga floristic divisions of Pechoro-Ilych State Reserve (Lavrenko et al. 1995). Earlier plant records for these divisions were occasional with 70 species known from Man-Pupu-Ner and 53 species from Middle Lyaga divisions (Ulle 2005), but no species lists were published to support these figures. Pechoro-Ilych Reserve is situated in Troitsko-Pechorsky District, Komi Republic on the western slope of the Urals. The Man-Pupu-Ner division covers mountain tundras and upper taiga belt, whereas Middle Lyaga division is fully located in the taiga elevational belt. The mobilised primary source (Seregin 2014) includes 325 species recorded in 2013 — 182 species in Man-Pupu-Ner and 143 species in Middle Lyaga. The centroid of the Man-Pupu-Ner division is located near the Reserve's cordon (coordinate uncertainty 8,500 m) and the centroid of the Middle Lyaga division is situated near the small winter hut (shelter) on the left bank of the Middle Lyaga River (coordinate uncertainty 10,000 m).

The Man-Pupu-Ner flora was fairly-well documented by herbarium vouchers, whereas a checklist for the Middle Lyaga division was primarily based upon vegetation relevés. The related occurrences from the MW GBIF-mediated dataset (Seregin 2021b) include 151 specimens — 131 specimens of 105 species from Man-Pupu-Ner (collector's numbers from U-166 to U-292) and 20 specimens of 19 species from Middle Lyaga (U-293 to U-311).

### 08. Utrish

This local flora (195 km^2^) stretches along the coastal zone of Abrau Peninsula from Sukko to Yuzhnaya Ozereyevka. It includes the Navagir Range, the westernmost spur of the Caucasus in Anapa and Novorossiysk Districts, Krasnodar Krai. The area shelters the relic ecosystems of arid sub-Mediterranean forests.

The first detailed floristic data on the flora of the Abrau Peninsula were published by Flerov and Flerov (1926). They indicated 991 species for the Anapa - Novorossiysk coastal area with 654 records documented by specimens, but their collections were entirely lost. Some of their records are doubtful and believed to be erroneous according to the recent studies of vascular flora of the NW Transcaucasus (Zernov 2000). Later, the flora of the Abrau Peninsula was studied during individual trips of a number of botanists, but it has never received special interest. Zernov (2000) reported 1,461 species for the whole territory of the North-Western Transcaucasus from Anapa to Tuapse (ca. 5,100 km^2^), based on herbarium specimens.

Tutors and students of the Biogeography Department, Moscow State University have been visiting annually the Utrish area for studies in field camps since 1997. A preliminary checklist of the area included 485 species, based upon collections and observations from 1997-1999 and was published as a brief manual for students (Syomina and Suslova 2000). Seregin and Suslova (2002) reported an additional 268 species and Seregin and Suslova (2007a) published 148 new records from the area. The preliminary checklist and two additions formed the basis of the large survey which listed 872 species (Seregin and Suslova 2007b). With 103 new records, the mobilised primary source (Suslova et al. 2015) is the most comprehensive checklist of the Utrish area. It includes 933 species recorded mainly from 2001-2010. The centroid of the area is located near the Malyi Utrish Marine Station with proposed coordinate uncertainty 17,000 m.

The Utrish flora is precisely documented by vouchers deposited in the MWG Herbarium (Herbarium of Biogeography Department, Faculty of Geography, Lomonosov Moscow State University). By 2015, there were 2,970 specimens from the Utrish area in MWG (Suslova et al. 2015), including 848 collections by the author made from 2001-2005 (collector's numbers from C-1 to C-517 in 2001, C-518 to C-712 in 2004, C-713 to C-848 in 2005), as well as other collections by department's students and staff. Unfortunately, there are no digitisation activities in this Herbarium at the moment.

### 09. Yasnaya Polyana

This small local flora (2.2 km^2^) was studied within the borders of the Leo Tolstoy memorial estate. It is situated in the broadleaved forest zone (temperate deciduous hardwood forests) in Shchekino District, Tula Oblast. The list of vascular plants from this location was never published as either a paper or a technical report. Currently, it includes 236 species recorded by the author in 2014 and 2017. The centroid of the flora is located near Tolstoy's tomb with proposed coordinate uncertainty 1,000 m.

The Yasnaya Polyana flora was not documented by vouchers during the author's field surveys, but some specimens from this location, collected by other botanists, can be found in TUL and MW GBIF-mediated datasets. For instance, 710 digitised specimens of 264 wild and cultivated species from Yasnaya Polyana are deposited at TUL Herbarium (Svetasheva and Seregin 2020, Svetasheva and Seregin 2021) and 67 digitised specimens of 57 species are available at MW Herbarium (Seregin 2021b).

### 10. Bogolyubovsky Lug

This small local flora (1.7 km^2^) was studied within the borders of the historical landscape complex "Bogolyubovsky Meadow - Church of the Intercession on the Nerl", a site with a legal status of the regional protected area. This is a large managed meadow, situated in the flood plain of the Klyazma River in the ecotone zone of mixed broadleaved and conifer forest near Bogolyubovo, Vladimir Oblast. The list of vascular plants from this location was never published as a paper, but was available online as a technical report (Seregin 2010). This report with 289 species, recorded by the author in 2010, was mobilised.

The Bogolyubovsky Lug flora was poorly documented by vouchers during the author's field surveys with only 11 specimens of ten species available in the MW Herbarium dataset (Seregin 2021b). The author's data from all Vladimir Oblast localities collected from 1999-2020 were fully integrated into a recently updated grid dataset (Seregin 2021a, Seregin 2021c) with coordinate uncertainty 7,000 m, an average distance between a grid square centroid and a grid square corner. The centroid of the Bogolyubovsky Lug flora is located in the middle of the actual area with proposed coordinate uncertainty 1,100 m.

### 11. Tsaritsyno

This small local flora (5.3 km^2^) was studied within the borders of the Tsaritsyno Museum-Estate, a site with a legal status of the regional protected area (as part of Tsaritsyno Nature Reserve). This is a large old park with a number of ponds, large lawns and managed meadows in the Southern Administrative Okrug, City of Moscow, situated in the ecotone zone of mixed broadleaved and conifer forests. The list of vascular plants from this location was never published as either a paper or a technical report. Currently, it includes 359 species recorded by the author in 2019.

The Tsaritsyno flora was not documented by herbarium vouchers during the author's field surveys, although a dataset of georeferenced photos made in 2019 was uploaded on iNaturalist. Thereby, photo vouchers of 773 observations of 337 species are available on GBIF (Ueda 2021) in line with the "Flora of Russia" initiative (Seregin et al. 2020).

### 12. Patakino

This small local flora (1.1 km^2^) was studied within the borders of the Patakinskaya Grove Nature Sanctuary, a site with a legal status of regional protected area. The area includes a broadleaved forest on a steep south-faced slope of the Klyazma River valley and an adjacent birch forest. The sanctuary is situated in the ecotone zone of mixed broadleaved and conifer forests near Patakino, Kameshkovsky District, Vladimir Oblast. The list of vascular plants from this location was never published as a paper, but was available online as a technical report (Seregin 2013). I mobilised this report with 312 species recorded in 2010.

The Patakino flora was poorly documented by vouchers during the author's field surveys with only 20 specimens of 19 species available in MW Herbarium dataset (Seregin 2021b). The author's data were fully integrated into a recently updated Vladimir Oblast grid dataset with coordinate uncertainty 7,000 m (see section "10. Bogolyubovsky Lug" for the details). The centroid of the Patakino flora is located in the middle of the actual area with proposed coordinate uncertainty 1,000 m.

## General description

### Purpose

The purpose of this dataset (Seregin 2021d) is to deliver to a wider audience in the format of GBIF-mediated data vast floristic materials collected by the author across various localities of European Russia from 2001-2019.

First of all, I selected for this dataset two thoroughly surveyed floras from small protected areas of Vladimir Oblast which have more precise georeferences than in the recently updated grid dataset (Seregin 2021a). For years, these two lists were available only as online technical reports. Floristic inventories of two other legally protected sites (Yasnaya Polyana in Tula Oblast and Tsaritsyno in the City of Moscow) were never published before, although these locations are popular amongst tourists. Another eight checklists have been published earlier in Russian, either as journal articles (Zaseki, Polibino, Khrenovoye, Troyeruchitsa, Man-Pupu-Ner and Middle Lyaga) or as chapters in thematic collections of research papers, a common genre of botanical writing in Russia (Ustya and Utrish).

Taxonomic data from these sources were mobilised, standardised and finally published as a GBIF-mediated dataset. It includes 5,309 species records (one species record per each local flora). These are either native or alien (fully naturalised and casual) species. Cultivated species were not recorded by the author. All records within one local flora have the same centroid coordinates and position accuracy (i.e. coordinate uncertainty in metres). See Table 1 for the details.

### Additional information

The nomenclature of the dataset follows the original mobilised sources. As a rule, they were based upon standard regional floras and checklists. Seregin and Goryainova (2003) followed Tzvelev (2000) for the Ustya local flora, Arkhangelsk Oblast. Seregin (2014) followed Lavrenko et al. (1995) for two local floras in Komi Republic. Both Seregin (2020) for the Troyeruchitsa local flora, Tver Oblast and Seregin (2013) for the Patakino local flora, Vladimir Oblast followed the nomenclature in Seregin (2012). Suslova et al. (2015) largely followed the standard flora by Zernov (2006) for the Utrish local flora, Krasnodar Krai.

Seregin (2010), Seregin (2011) and Seregin (2015) did not provide any general taxonomic reference, but their backbone was synchronised with a standard flora of Vladimir Oblast by Seregin (2012). The same reference was used for the unpublished checklist of the Yasnaya Polyana local flora. A taxonomic backbone for the unpublished checklist of the Tsaritsyno local flora, City of Moscow was downloaded in 2019 from iNaturalist, which follows POWO taxonomy (POWO 2021).

## Geographic coverage

### Description

**Figure 1.**
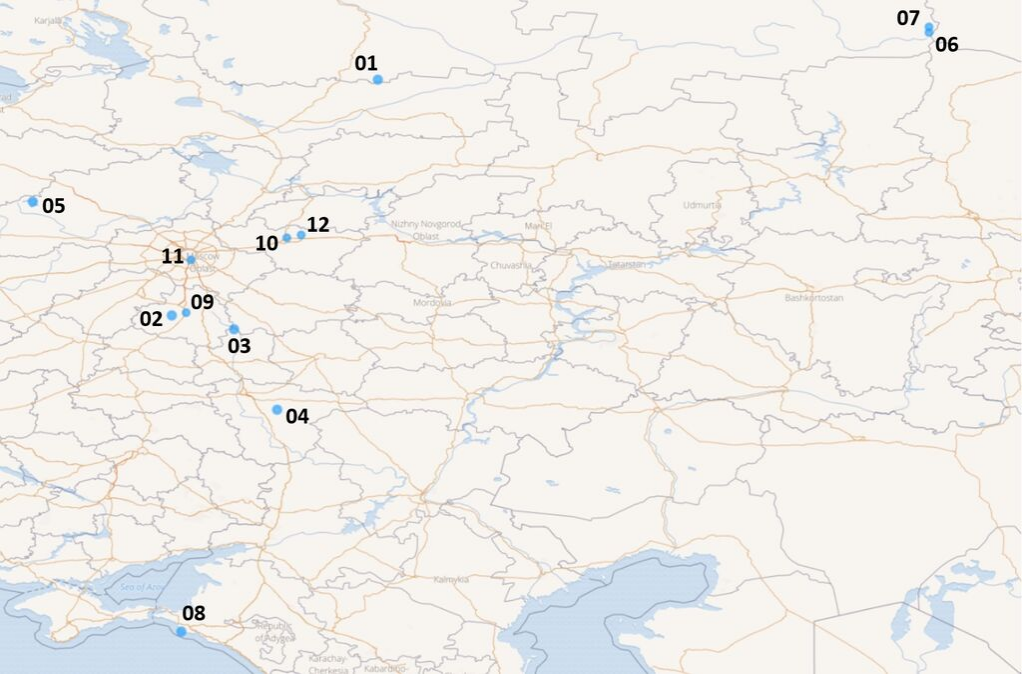
Distribution of the sampled local floras across European Russia: **01** - Ustya, Arkhangelsk Oblast; **02** - Zaseki, Tula Oblast; **03** - Polibino, Lipetsk Oblast; **04** - Khrenovoye, Voronezh Oblast; **05** - Troyeruchitsa, Tver Oblast; **06** - Man-Pupu-Ner, Komi Republic; **07** - Middle Lyaga, Komi Republic; **08** - Utrish, Krasnodar Krai; **09** - Yasnaya Polyana, Tula Oblast; **10** - Bogolyubovsky Lug, Vladimir Oblast; **11** - Tsaritsyno, City of Moscow; **12** - Patakino, Vladimir Oblast. Source: Seregin (2021d).

Twelve local floras are situated in various places across European Russia. Their locations are given on the map (Fig. 1). Some additional information for each locality is given in the introduction.

Brief general information about the studied areas is given in Table 1. Typical plant communities of the locations are given in the series of photographs (Figs 2, 3, 4, 5, 6, 7, 8, 9, 10, 11, 12, 13).

### Coordinates

44 and 63 Latitude; 33 and 60 Longitude.

## Taxonomic coverage

### Description

According to the GBIF taxonomic backbone, the dataset covers 1,806 species, 669 genera and 127 families of tracheophytes.

Phylum Tracheophyta.

## Temporal coverage

### Notes

2001 to 2019

## Usage licence

### Usage licence

Creative Commons Public Domain Waiver (CC-Zero)

## Data resources

### Data package title

Twelve local floras of European Russia

### Resource link

https://doi.org/10.15468/35rwhv

### Alternative identifiers

https://www.gbif.org/dataset/78d38015-7cb8-425a-a328-cac5634f480e

### Number of data sets

1

### Data set 1.

#### Data set name

Twelve local floras of European Russia

#### Data format

GBIF annotated archive

#### Number of columns

47

#### Download URL

https://www.gbif.org/occurrence/download?dataset_key=78d38015-7cb8-425a-a328-cac5634f480e

#### Description

Twelve local floras of European Russia studied and documented from 2001-2019 by Dr. Alexey P. Seregin: Ustya (Arkhangelsk Oblast), Zaseki (Tula Oblast), Polibino (Lipetsk Oblast), Khrenovoye (Voronezh Oblast), Troyeruchitsa (Tver Oblast), Man-Pupu-Ner (Komi Republic), Middle Lyaga (Komi Republic), Utrish (Krasnodar Krai), Yasnaya Polyana (Tula Oblast), Bogolyubovsky Lug (Vladimir Oblast), Tsaritsyno (City of Moscow) and Patakino (Vladimir Oblast).

**Data set 1.** 

Column label	Column description
occurrenceID	An identifier for the Occurrence (as opposed to a particular digital record of the occurrence). A variable constructed from a combination of three identifiers in the record that will most closely make the occurrenceID globally unique (datasetID + ID of a local flora + ID of a record within the local flora). For example, "urn:lsid:biocol.org:col:15550:10:01:100".
dcterms:type	The nature or genre of the resource. A constant ("Dataset").
dcterms:modified	The most recent date-time on which the resource was changed. A constant ("2021-08-11").
dcterms:language	A language of the resource. A constant ("en" = English)
dcterms:license	A legal document giving official permission to do something with the resource. A constant ("http://creativecommons.org/licenses/by/4.0/legalcode").
dcterms:rightsHolder	A person or organisation owning or managing rights over the resource. A constant ("Moscow State University").
dcterms:accessRights	Information about who can access the resource or an indication of its security status. A constant ("Use under CC BY 4.0").
institutionID	An identifier for the institution having custody of the object(s) or information referred to in the record. A constant ("http://grbio.org/institution/moscow-stateuniversity" for the Moscow Sate University).
collectionID	An identifier for the collection or dataset from which the record was derived. A constant ("urn:lsid:biocol.org:col:15550" for the Moscow University Herbarium).
datasetID	An identifier for the set of data. May be a global unique identifier or an identifier specific to a collection or institution. A constant ("urn:lsid:biocol.org:col:15550:10").
institutionCode	The name (or acronym) in use by the institution having custody of the object(s) or information referred to in the record. A constant ("Moscow State University").
datasetName	The name identifying the dataset from which the record was derived. A constant ("Twelve local floras of European Russia").
ownerInstitutionCode	The name (or acronym) in use by the institution having ownership of the object(s) or information referred to in the record. A constant ("Moscow State University").
basisOfRecord	The specific nature of the data record - a subtype of the dcterms:type. A constant ("HumanObservation").
informationWithheld	Additional information that exists, but that has not been shared in the given record. A variable. For example, "Associated ecological data and frequency estimate", "Associated ecological data, frequency estimate and a year of the record", "Voucher reference".
catalogNumber	An identifier (preferably unique) for the record within the dataset or collection. A variable. For example, "Ustya:100" (a species with a number #100 within the list of Ustya local flora).
recordedBy	A list (concatenated and separated) of names of people, groups or organisations responsible for recording the original occurrence. A variable. For example, "Alexey P. Seregin".
occurrenceStatus	A statement about the presence or absence of a taxon at a location. A constant ("present").
associatedReferences	A list (concatenated and separated) of identifiers (publication, bibliographic reference, global unique identifier, URI) of literature associated with the Occurrence. A variable. For example, "Seregin, A.P. (2014): Floristic records from Man-Pupu-Nyor Range and adjacent paths (Pechora-Ilych Reserve, Republic of Komi, Russia). Fitoraznoobrazie Vostochnoy Evropy 8 (2), 97–105 (in Russian, with English abstract)".
eventDate	The date or interval during which an event occurred. For occurrences, this is the date when the event was recorded. A variable.
higherGeography	A list (concatenated and separated) of geographic names less specific than the information captured in the locality term. A variable (for example, "Europe | Russian Federation | Vladimir Oblast").
continent	The name of the continent in which the location occurs. A constant ("Europe").
country	The name of the country or major administrative unit in which the location occurs. A constant ("Russian Federation").
countryCode	The standard code for the country in which the location occurs. A constant ("RU").
stateProvince	The name of the next smaller administrative region than country (state, province, canton, department, region etc.) in which the location occurs. A variable (for example, "Krasnodar Krai").
locality	The specific description of the place. This term may contain information modified from the original to correct perceived errors or standardise the description. A variable (twelve options: "Bogolyubovsky Lug", "Khrenovoye", "Man-Pupu-Ner", "Middle Lyaga", "Patakino", "Polibino", "Troyeruchitsa", "Tsaritsyno", "Ustya", "Utrish", "Yasnaya Polyana", "Zaseki").
decimalLatitude	The geographic latitude (in decimal degrees, using the spatial reference systemgiven in geodeticDatum) of the geographic centre of a location. A variable (latitude of a local flora centroid).
decimalLongitude	The geographic longitude (in decimal degrees, using the spatial reference systemgiven in geodeticDatum) of the geographic centre of a location. A variable (longitude of a local flora centroid).
geodeticDatum	The ellipsoid, geodetic datum or spatial reference system (SRS) upon which the geographic coordinates given in decimalLatitude and decimalLongitude are based. A constant ("WGS84").
coordinateUncertaintyInMeters	The horizontal distance (in metres) from the given decimalLatitude and decimalLongitude describing the smallest circle containing the whole of the location. A variable. For example, "16000" in Khrenovoye local flora.
coordinatePrecision	A decimal representation of the precision of the coordinates given in the decimalLatitude and decimalLongitude. A constant ("0.0001").
georeferencedBy	A list (concatenated and separated) of names of people, groups or organisations who determined the georeference (spatial representation) of the location. A constant ("Alexey P. Seregin").
georeferencedDate	The date on which the Location was georeferenced. A constant ("2021-08-10").
georeferenceSources	A list (concatenated and separated) of maps, gazetteers, or other resources used to georeference the Location, described specifically enough to allow anyone in the future to use the same resources. A constant ("https://yandex.ru/maps/").
identifiedBy	A list (concatenated and separated) of names of people, groups or organisations who assigned the Taxon to the subject. A variable (for example, "Alexey P. Seregin").
scientificName	The full scientific name, with authorship and date information, if known. A variable (for example, "Scirpus sylvaticus L.").
nameAccordingTo	For taxa that result from identifications, a reference to the keys, monographs, experts and other sources should be given. A variable.
kingdom	The full scientific name of the kingdom in which the taxon is classified. A constant ("Plantae").
phylum	The full scientific name of the phylum or division in which the taxon is classified. A constant ("Tracheophyta").
genus	The full scientific name of the genus in which the taxon is classified. A variable (for example, "Scirpus").
specificEpithet	The name of the first or species epithet of the scientificName. A variable (for example, "sylvaticus").
infraspecificEpithet	The name of the lowest or terminal infraspecific epithet of the scientificName, excluding any rank designation. A variable (for example, "ucrainicum").
taxonRank	The taxonomic rank of the most specific name in the scientificName. A variable (four options: "species", "subspecies", "genus", "speciesAggregate").
scientificNameAuthorship	The authorship information for the scientificName formatted according to the conventions of the applicable nomenclaturalCode. A variable (for example, "(L.) Roem. et Schult.").
nomenclaturalCode	The nomenclatural code (or codes in the case of an ambiregnal name) under which the scientificName is constructed. A constant ("International Code of Nomenclature for algae, fungi and plants").
taxonomicStatus	The status of the use of the scientificName as a label for a taxon. A constant ("accepted").
identificationQualifier	A brief phrase or a standard term to express the determiner's doubts about the Identification. A variable (two options: "sensu lato", "sensu stricto").

## Figures and Tables

**Figure 2.**
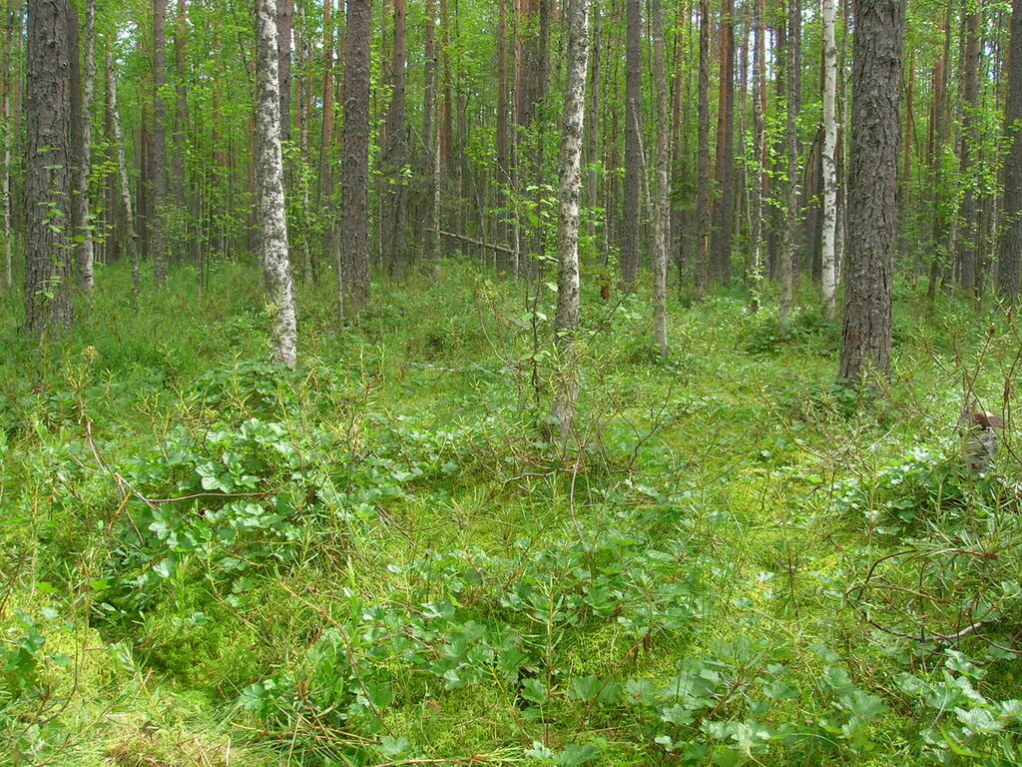
Typical plant communities of the Ustya local flora (Arkhangelsk Oblast, Russia): small peat bog (Oxycocco-Sphagnetea) in taiga forest (Vaccinio-Piceetea). Photo by Irina P. Seregina (2006).

**Figure 3.**
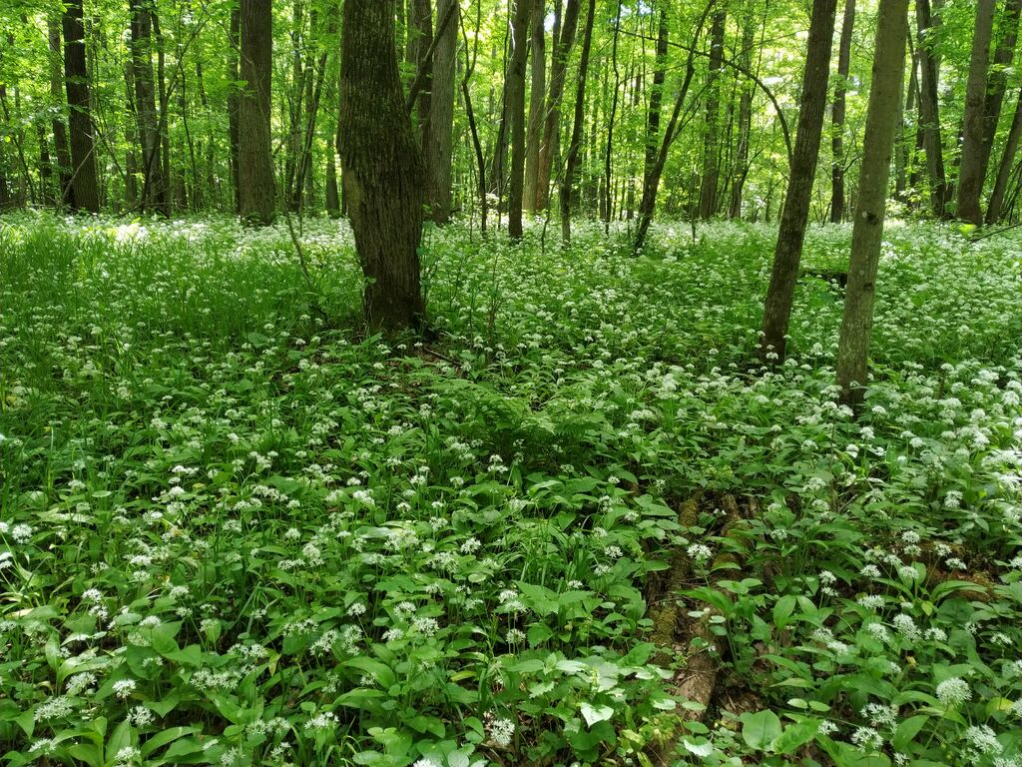
Typical plant communities of the Zaseki local flora (Tula Oblast, Russia): broadleaved forest (Querco-Fagetea) with *Alliumursinum* L. Photo by the author (2021).

**Figure 4.**
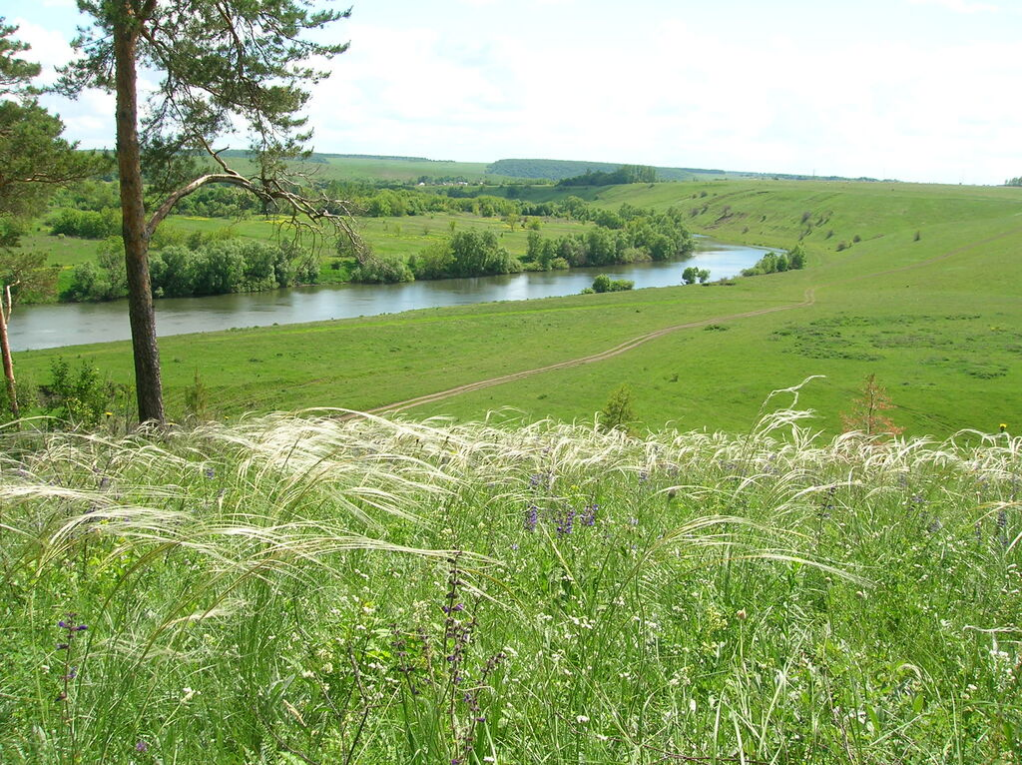
Typical plant communities of the Polibino local flora (Lipetsk Oblast, Russia): steppe (Festuco-Brometea) with *Stipapennata* L. Photo by the author (2006).

**Figure 5.**
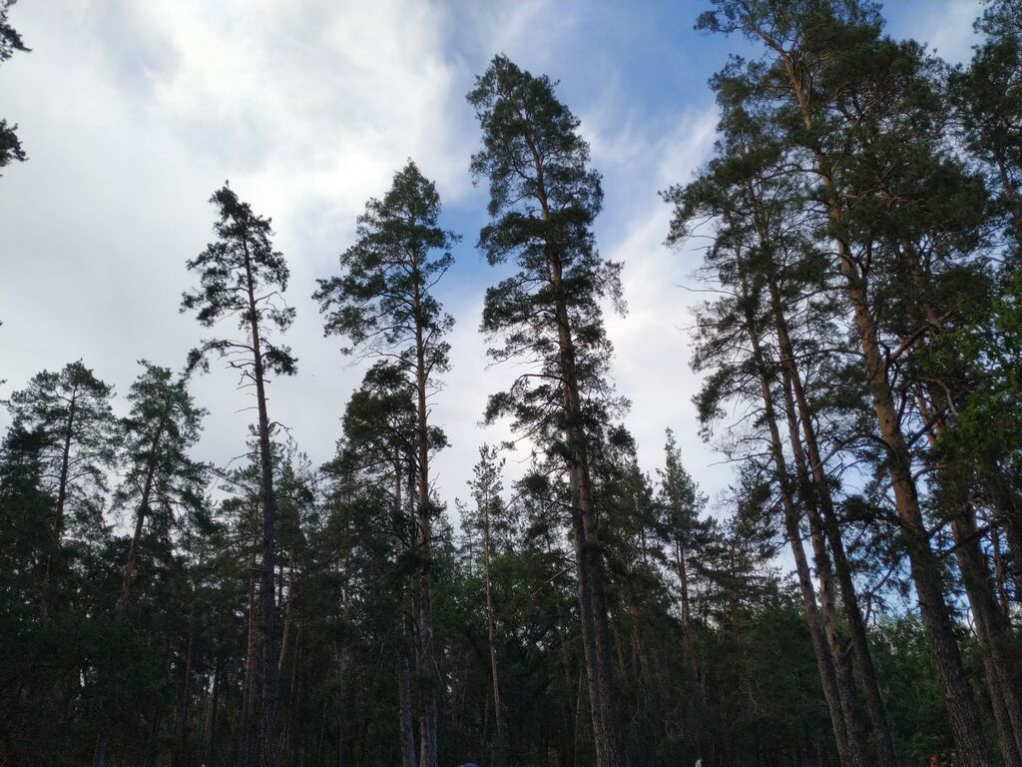
Typical plant communities of the Khrenovoye local flora (Voronezh Oblast, Russia): the edge of thermophilous pine forest (Pyrolo-Pinetea). Photo by the author (2021).

**Figure 6.**
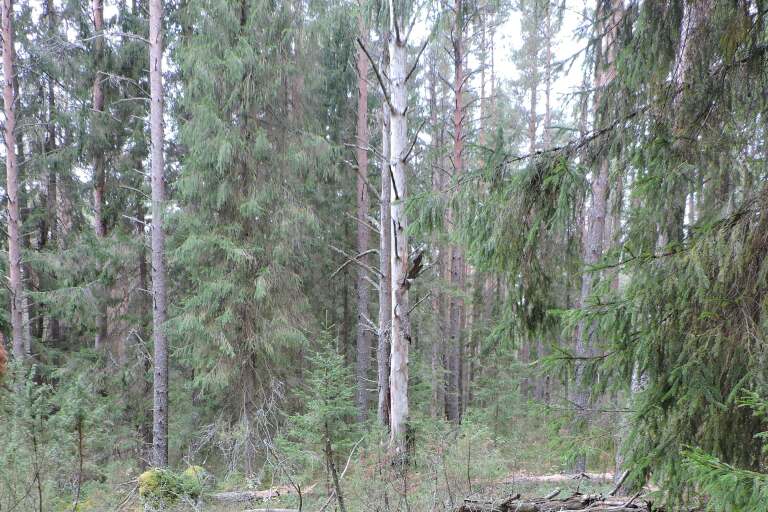
Typical plant communities of the Troyeruchitsa local flora (Tver Oblast, Russia): taiga forest (Vaccinio-Piceetea). Photo by the author (2016).

**Figure 7.**
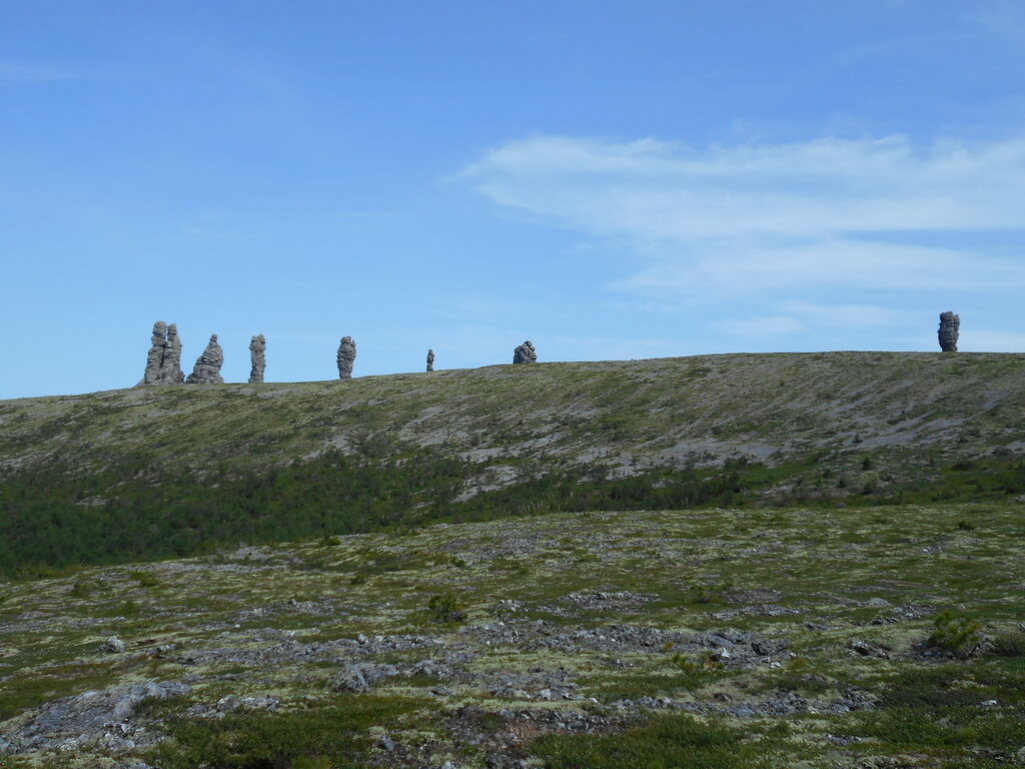
Typical plant communities of the Man-Pupu-Ner local flora (Komi Republic, Russia): tundra (Loiseleurio–Vaccinietea) above timber-line, 700-750 m a.s.l. Photo by the author (2013).

**Figure 8.**
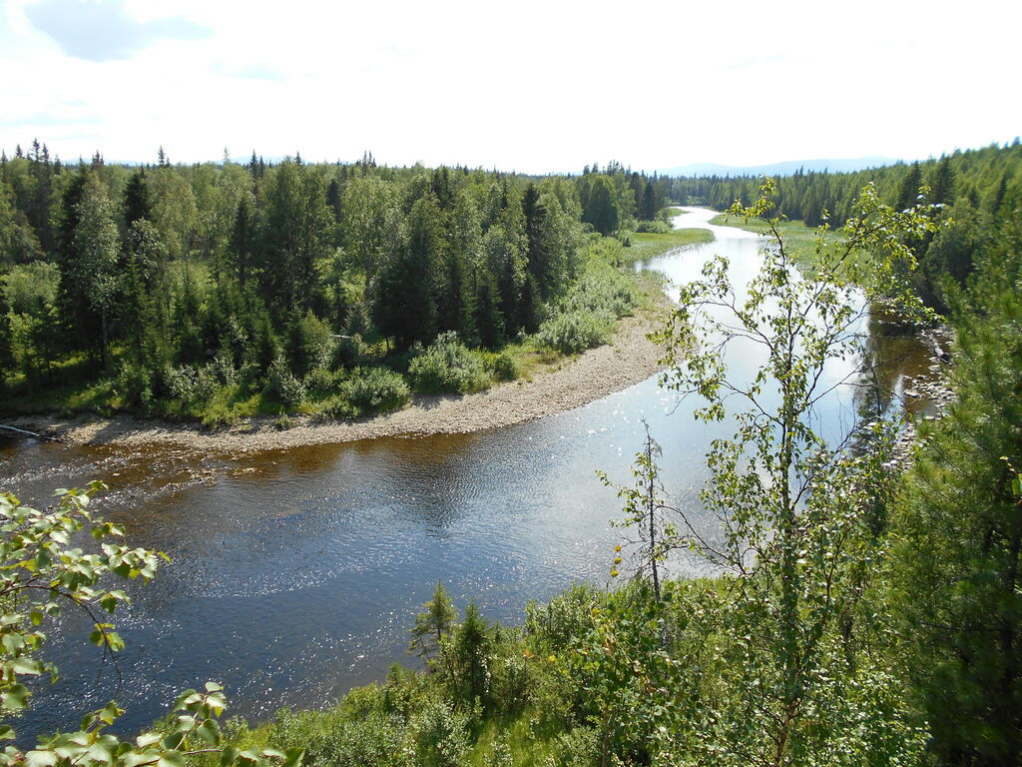
Typical plant communities of the Middle Lyaga local flora (Komi Republic, Russia): river pebbles enframed by taiga forest (Vaccinio-Piceetea), 230 m a.s.l. Photo by the author (2013).

**Figure 9.**
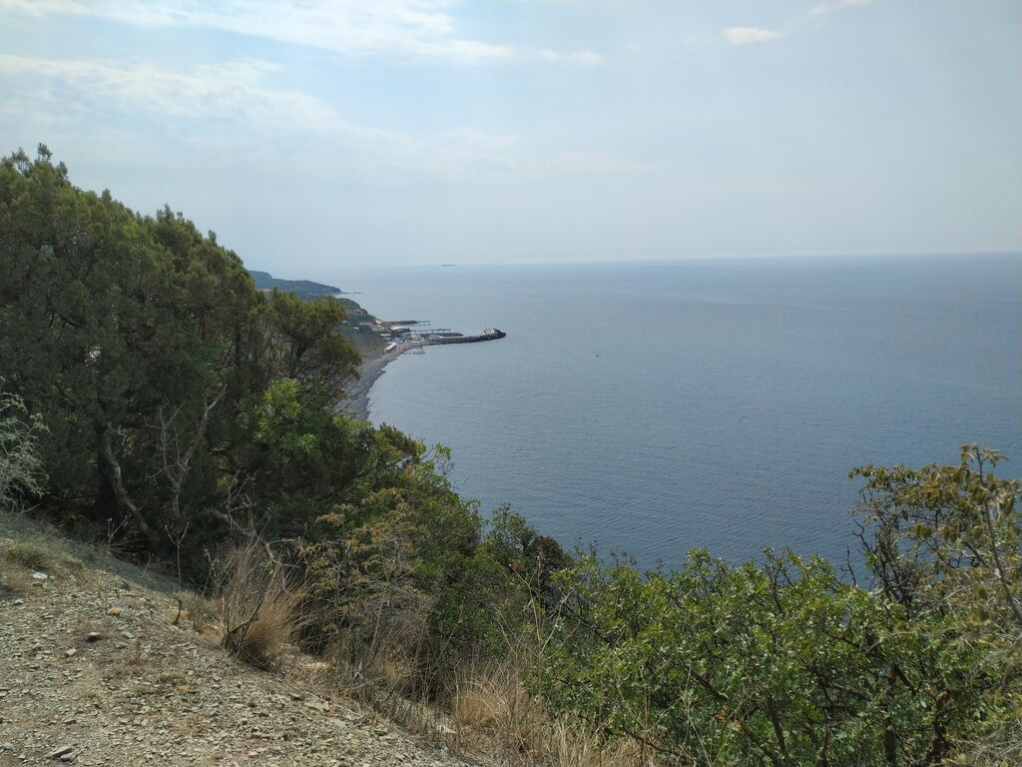
Typical plant communities of the Utrish local flora (Krasnodar Krai, Russia): xeric stands of *Juniperusexcelsa* M.Bieb. along the Black Sea Coast. Photo by the author (2020).

**Figure 10.**
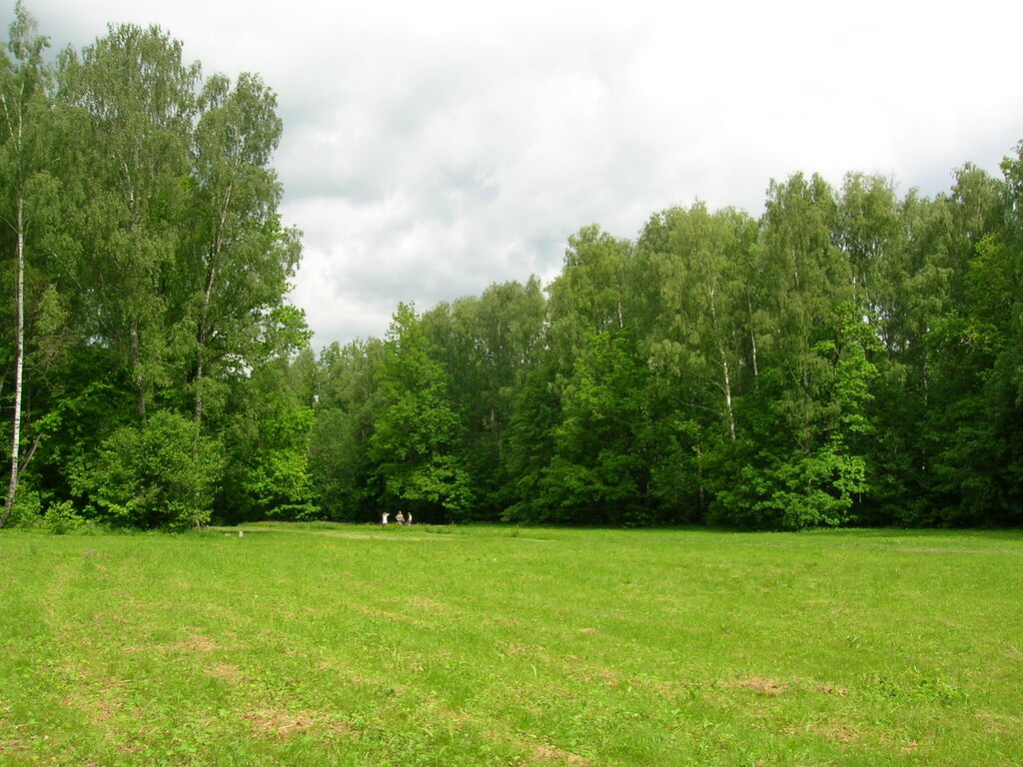
Typical plant communities of the Yasnaya Polyana local flora (Tula Oblast, Russia): managed meadow (Molinio-Arrhenatheretea) on the edge of broadleaved forest (Querco-Fagetea). Photo by the author (2006).

**Figure 11.**
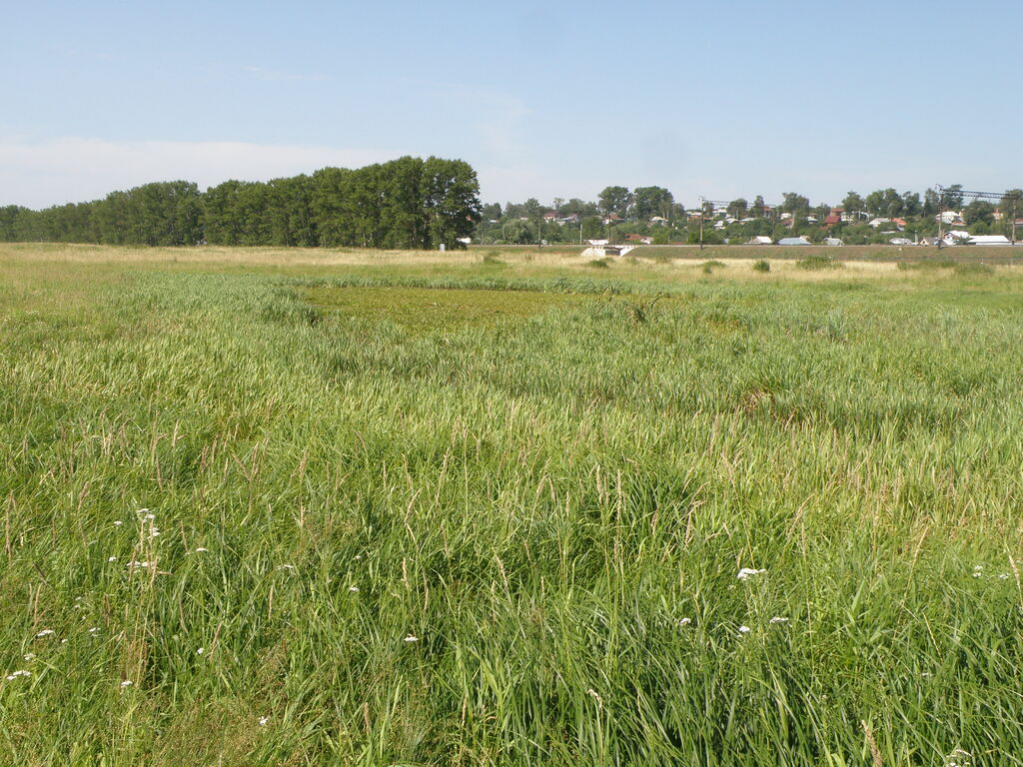
Typical plant communities of the Bogolyubovsky Lug local flora (Vladimir Oblast, Russia): *Stratioitesaloides* L. community (Lemnetea) surrounded by Phragmito-Magnocaricetea swamp and Molinio-Arrhenethretea meadows on flood plain of the Klyazma River. Photo by the author (2010).

**Figure 12.**
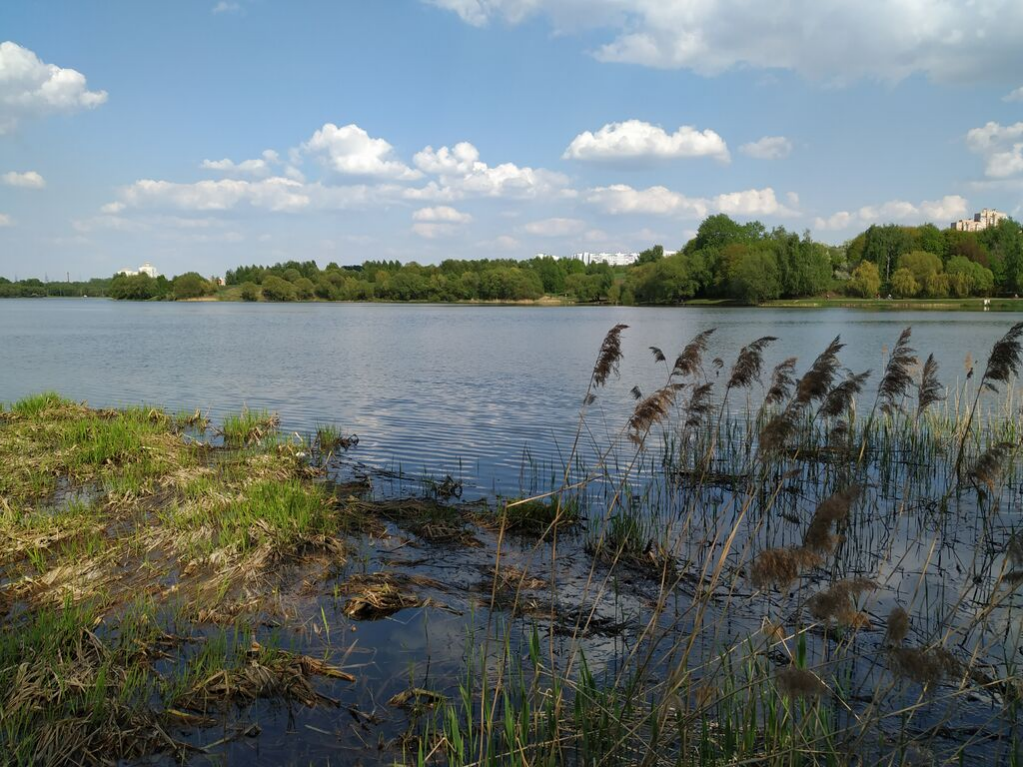
Typical plant communities of the Tsaritsyno local flora (City of Moscow, Russia): reed communities (Phragmito-Magnocaricetea) on the bank of a water reservoir. Photo by the author (2019).

**Figure 13.**
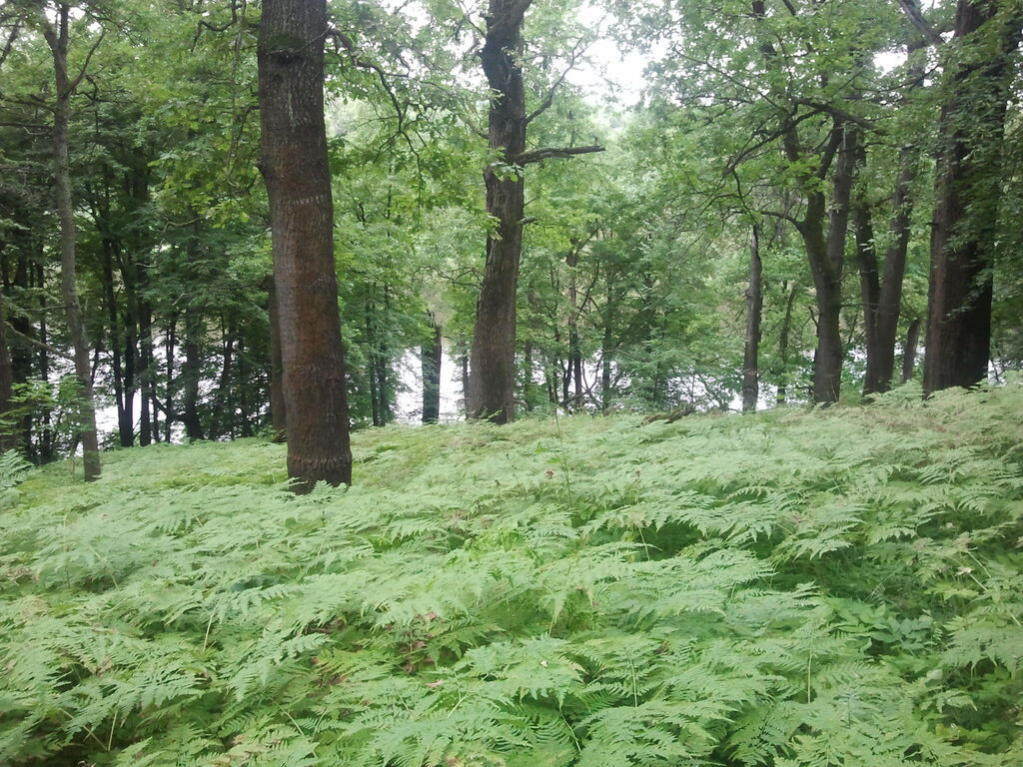
Typical plant communities of the Patakino local flora (Vladimir Oblast, Russia): broadleaved forest (Querco-Fagetea) dominated by *Pteridiumpinetorum* C.N. Page & R.R. Mill. Photo by the author (2013).

**Table 1.** General information on local floras.

Number	Local flora	Region	Number of taxa	Latitude of the centroid	Longitude of the centroid	Coordinate uncertainty in metres	Area in square kilometres	Mobilised source
01	Ustya	Arkhangelsk Oblast	543	60.8956	43.2149	21,000	1,500	Seregin and Goryainova (2003)
02	Zaseki	Tula Oblast	593	53.9715	37.1809	8,000	60	Seregin (2011), Seregin (2015)
03	Polibino	Lipetsk Oblast	553	53.4942	38.9942	7,000	70	Seregin (2011), Seregin (2015)
04	Khrenovoye	Voronezh Oblast	665	51.1968	40.2515	15,000	200	Seregin (2015)
05	Troyeruchitsa	Tver Oblast	501	57.2454	33.1024	3,000	10	Seregin (2020)
06	Man-Pupu-Ner	Komi Republic	182	62.2479	59.3074	8,500	ca. 300	Seregin (2014)
07	Middle Lyaga	Komi Republic	143	62.3760	59.2656	10,000	ca. 300	Seregin (2014)
08	Utrish	Krasnodar Krai	933	44.7057	37.4695	17,000	195	Suslova et al. (2015)
09	Yasnaya Polyana	Tula Oblast	236	54.0776	37.5197	1,000	2.2	previously unpublished
10	Bogolyubovsky Lug	Vladimir Oblast	289	56.1918	40.5529	1,100	1.7	Seregin (2010)
11	Tsaritsyno	City of Moscow	359	55.6211	37.6826	2,000	5.3	previously unpublished
12	Patakino	Vladimir Oblast	312	56.2654	40.8993	1,000	1.1	Seregin (2013)
